# The First Research in Your Career: How to Use your Resources as Productively as Possible

**DOI:** 10.1192/j.eurpsy.2022.204

**Published:** 2022-09-01

**Authors:** N. Sartorius

**Affiliations:** AIMHP, Association For The Improvement Of Mental Health Programs, Genève, Switzerland

## Abstract

The first engagement in research in your career This presentation will argue that in addition to considering the scientific interest of a topic presented for research it is important to consider other criteria before engaging in a study. These include the place of the study, the team which will be engaged in the work, the ownership of the data which will be produced, the duration of the study and other matters. The presentation will also discuss the amount of time that should be given to scientific research early in one’s career and the nature of the gain that engagement in research can offer for one’s development and career.

**Disclosure:**

No significant relationships.

